# Functional and clinical analysis of five *EDA* variants associated with ectodermal dysplasia but with a hard-to-predict significance

**DOI:** 10.3389/fgene.2022.934395

**Published:** 2022-07-18

**Authors:** Sare Gökdere, Holm Schneider, Ute Hehr, Laure Willen, Pascal Schneider, Sigrun Maier-Wohlfart

**Affiliations:** ^1^ Department of Pediatrics, Center for Ectodermal Dysplasias, University Hospital Erlangen, Erlangen, Germany; ^2^ Center for Human Genetics, Regensburg, Germany; ^3^ Department of Biochemistry, University of Lausanne, Lausanne, Switzerland

**Keywords:** X-linked hypohidrotic ectodermal dysplasia, ectodysplasin A, variants of uncertain significance, *in silico* analysis, functional studies, serum EDA concentration, genotype-phenotype correlation

## Abstract

Deficiency of ectodysplasin A1 (EDA1) due to variants of the gene *EDA* causes X-linked hypohidrotic ectodermal dysplasia (XLHED), a rare genetic condition characterized by abnormal development of ectodermal structures. XLHED is defined by the triad of hypotrichosis, hypo- or anhidrosis, and hypo- or anodontia. Anhidrosis may lead to life-threatening hyperthermia. A definite genetic diagnosis is, thus, important for the patients’ management and amenability to a novel prenatal treatment option. Here, we describe five familial EDA variants segregating with the disease in three families, for which different prediction tools yielded discordant results with respect to their significance. Functional properties *in vitro* and levels of circulating serum EDA were compared with phenotypic data on skin, hair, eyes, teeth, and sweat glands. EDA1-Gly176Val, although associated with relevant hypohidrosis, still bound to the EDA receptor (EDAR). Subjects with EDA1-Pro389LeufsX27, -Ter392GlnfsX30, -Ser125Cys, and an EDA1 splice variant (c.924+7A > G) showed complete absence of pilocarpine-induced sweating. EDA1-Pro389LeufsX27 was incapable of binding to EDAR and undetectable in serum. EDA1-Ter392GlnfsX30, produced in much lower amounts than wild-type EDA1, could still bind to EDAR, and so did EDA1-Ser125Cys that was, however, undetectable in serum. The *EDA* splice variant c.924+7A > G resulted experimentally in a mix of wild-type EDA1 and EDA molecules truncated in the middle of the receptor-binding domain, with reduced EDA serum concentration. Thus, *in vitro* assays reflected the clinical phenotype in two of these difficult cases, but underestimated it in three others. Absence of circulating EDA seems to predict the full-blown phenotype of XLHED, while residual EDA levels may also be found in anhidrotic patients. This indicates that unborn subjects carrying variants of uncertain significance could benefit from an upcoming prenatal medical treatment even if circulating EDA levels or tests *in vitro* suggest residual EDA1 activity.

## Introduction

X-linked hypohidrotic ectodermal dysplasia (XLHED; MIM #305100) is a rare genetic condition affecting the normal development of ectodermal derivatives, such as skin, teeth, hair, and eccrine glands. This is reflected by three characteristic symptoms, namely, hypotrichosis, hypo- or anodontia, and hypo- or anhidrosis, the latter of which may lead to life-threatening hyperthermia, especially in early childhood (Blüschke et al., 2010). Many patients also display other abnormalities, such as chronic skin issues, recurrent respiratory problems, and ophthalmic pathologies (Clarke et al., 1987; Itin and Fistarol, 2004; Kaercher 2004; Dietz et al., 2013). XLHED is caused by pathogenic variants of the X-chromosomal ectodysplasin A gene (*EDA*; MIM *300451; NM_001399) encoding the transmembrane protein ectodysplasin A (EDA; Kere et al., 1996). The protein consists of 391 amino acids and contains an intracellular domain (ICD), a transmembrane domain (TM), and an extracellular domain (ECD) with three regions considered to be mutational hotspots, namely, a furin cleavage site (FUR, consisting of two overlapping consensus sequences), a collagen-like domain (COL), and a C-terminal tumor necrosis factor homology (TNF) domain responsible for receptor binding. N-terminal of the COL, a short heparan sulfate proteoglycan binding region (HSPG) has been reported to restrict the distribution of endogeneous EDA; however, its physiological function is not fully understood and no disease-causing variants within this region have been published yet (Swee et al., 2009; Podzus et al., 2017). Pathogenic variants in the FUR inhibit the essential proteolytic cleavage of EDA, while those within the COL may hamper the necessary multimerization of the TNF domain. Two EDA splice variants, EDA1 and EDA2, are known, which interact with distinct receptors. Binding of EDA1 to its receptor EDAR can be disturbed by pathogenic variants within the TNF region (Schneider et al., 2001). While null mutations normally lead to a complete loss of protein function and the full-blown clinical picture; hypomorphic variants are associated with residual EDA activity and milder phenotypes (Burger et al., 2014). Variants of the gene *EDA* may not only be responsible for XLHED but also for non-syndromic tooth agenesis (Mues et al., 2009).

So far, therapeutic options have been limited to preventive measures and symptomatic treatments like avoiding heat, external cooling devices, and dental implants. However, a novel therapeutic approach to XLHED based on a recombinant EDA molecule (a fusion protein consisting of the receptor-binding domain of EDA1 and the Fc domain of human IgG1) proved successful in animal models of the disease (Gaide and Schneider 2003; Hermes et al., 2014; Margolis et al., 2019), and intra-amniotic administration of this replacement protein has been shown to prevent most XLHED symptoms in human patients (Schneider et al., 2018; Körber et al., 2020). A clinical trial to evaluate the prenatal protein replacement is currently being conducted (EudraCT No. 2021-002532-23). One of the inclusion criteria for this trial is the diagnosis of XLHED in the yet unborn patient by tooth germ sonography or genetic testing, requiring assessment of the familial *EDA* variant as pathogenic. However, classification of sequence variants of uncertain significance (VUS), according to the guidelines of the American College of Medical Genetics and Genomics (ACMG) as pathogenic (class 5), likely pathogenic (class 4), of uncertain significance (class 3), likely benign (class 2), or benign (class 1) is not always unambiguous (Richards et al., 2015). Evidence criteria for categorization might be based on *in silico* prediction tools, database and literature research, segregation analysis, allele frequencies, and/or functional *in vitro* assays.

This study aimed to investigate the impact of *EDA* variants of yet unclear significance in subjects with clinical manifestations of XLHED. Reliable classification of the familial variant is an essential requirement for offering a prenatal drug therapy to a pregnant carrier woman.

## Subjects and methods

### Study design and patients

In this monocentric observational study, five familial cases of XLHED with *EDA* variants of unknown significance were investigated. All adult patients gave written informed consent to the use of their blood or DNA for molecular analysis. In the case of minors, parental consent was obtained. The study was approved by the ethics committee of the University Erlangen-Nürnberg and conducted according to the principles of the Declaration of Helsinki.

### Assessment of XLHED-related phenotypic features

XLHED-related issues (including general health, heat intolerance, dentition, skin, and hair anomalies) were assessed by quantitative patient surveys and physical examinations. If possible, patients’ ability to perspirate was investigated by quantification of pilocarpine-induced sweating on the forearms (in an area of 57 mm^2^) using the Wescor 3700 device (Wescor, Logan, United States) and determination of palmar sweat duct densities *via* reflectance confocal laser-scanning microscopy with the VivaScope 1,500 (Caliber Imaging & Diagnostics, New York, United States), both as described before (Burger et al., 2014). The total number of sweat ducts is determined early in life, which means that the sweat gland density decreases with an increasing skin surface (adults have about 300–500/cm^2^, while newborns normally show more than 1,000/ cm^2^; Montagna and Parakkal, 1974).

### DNA analysis

DNA isolation from peripheral blood, polymerase chain reaction, and subsequent Sanger sequencing were performed as described previously (Wohlfart et al., 2016). Specific oligonucleotide primer sequences and thermal cycling conditions for the detection of *EDA* variants are available upon request. The software programs Sequencher 4.8 (Gene Codes Corporation, Ann Arbor, Michigan, United States) and ChromasPro 2.1.9 (Technelysium Pty Ltd., South Brisbane, Australia) were used for analysis of the electropherograms. The online prediction algorithms MutationTaster (Schwarz et al., 2014), NNSplice (Reese et al., 1997), PROVEAN (Choi et al., 2012), PolyPhen 2.0 (Adzhubei et al., 2010), SIFT (Kumar et al., 2009), and FATHMM (Shihab et al., 2013) were used for assessment of the variants. Database and literature research were performed using the Human Gene Mutation Database (HGMD), the Genome Aggregation Database (gnomAD), and PubMed. The variants were included in the Leiden Open Variation Database (https://www.lovd.nl/).

### Multi-gene panel sequencing

In one case, multi-gene panel sequencing was conducted. Coding exons and flanking exon–intron boundaries of selected ED-associated genes (*EDA*, *EDAR*, *EDARADD*, *TP63*, *WNT10A*, *CDH3*, *EDA2R*, *GJB6*, *IKBKG*, *IFT122*, *IFT43*, *KRT74*, *KRT85*, *MSX1*, *NFKBIA*, *PIGL*, *PVRL4*, *TRAF6*, *WDR19*, and *WDR35*) were analyzed by massive parallel sequencing using Nextera Enrichment (Illumina NextSeq; 2 × 150 bp paired-end run) and reads aligned to the human reference genome hg 19. Bioinformatic analysis and variant assessment were performed as described before according to the ACMG guidelines (Hinreiner et al., 2018).

### Antibodies, proteins, and cell culture

Antibodies anti-Flag M2 (Sigma Aldrich F3165) and anti-Flag coupled to agarose (Sigma Aldrich A2220) were used. Anti-hEDA mAb mouse IgG1 Renzo-2 (anti-EDA) was produced by hybridoma cells and affinity-purified on protein G-Sepharose (Cytiva 17,061,805). Renzo-2 is also available commercially (Enzo Life Sciences, ALX-804-839-C100). EDAR-Fc was produced by a stable clone of HEK cells and affinity-purified on protein A-Sepharose essentially as described (Schneider, 2000). EDA proteins were expressed by transient transfections in HEK 293T cells with the polyethyleneimide method, then placed in serum-free OptiMEM medium for 7 days, after which time cells and conditioned supernatants were harvested (Schneider et al., 2014). AD293 cells were cultured in Dulbecco’s modified Eagle’s medium with penicillin/streptomycin and 10% fetal calf serum (FCS). The cells were modified by (co-)transfection using standard transfection kits (K2 Transfection System, Biontex, Munich/Laim, Germany; Lipofectamine 3000 reagent, Thermo Fisher Scientific, Waltham, United States).

### Molecular cloning

Most of the EDA vector constructs were generated as described previously (Schneider 2000; Schneider et al., 2001). The vector pSPL3 which contains a small artificial gene (including a SV40 promoter, an exon–intron–exon sequence with splice donor and acceptor sites, and a late polyadenylation signal) was used for an investigation of the potential effects of the splice site variants by an exon trapping assay. For this purpose, the genomic sequences of interest were inserted into the multiple cloning site of this artificial gene to create a mini-gene expression construct. In most instances, experiments were conducted as described in the publications of Tompson and Young (2017), who provided us the pSPL3 vector, and Liu et al. (2018). Site-directed mutagenesis was performed in order to introduce the relevant variant into the particular wild-type (WT) gene sequence (QuikChange II Site-Directed Mutagenesis Kit, Agilent Technologies, Santa Clara, California, United States). A detailed list of plasmids used in the study and Sanger sequencing results of the different variants can be found in the Supplementary Table S1 and Supplementary Figure S2. Note that only exons present in the cDNA of *EDA* were numbered. The exon contained in intron 1 that is present in shorter splice variants but absent in the coding sequence of *EDA* was not counted.

### RNA isolation and reverse transcription

After transfection experiments, total RNA was extracted from the cells using TRIzol™ reagent (Thermo Fisher Scientific, Waltham, United States) and a standard protocol for RNA isolation. cDNA was synthesized by reverse transcription RT-PCR with the Moloney murine leukemia virus reverse transcriptase system (Promega GmbH, Mannheim, Germany), according to the manufacturer’s instructions.

### AlphaLISA immunoassay

EDA protein quantification in blood serum was performed in duplicate by AlphaLISA as described before (Podzus et al., 2017). In brief, samples in microplates were incubated for 1 h at room temperature with acceptor beads coupled to anti-EDA mAb EctoD2 and with biotinylated anti-EDA mAb EctoD3. Thereafter, streptavidin-coupled beads were added and incubated for 15 min. Plates were read with an Enspire plate reader (PerkinElmer, Schwerzenbach, Switzerland). A standard curve of recombinant Fc-EDA1 (provided by Edimer Pharmaceuticals) diluted in assay buffer or in serum from an adult male subject with the full-blown phenotype of XLHED was monitored in parallel.

### Co-immunoprecipitation and Western blot

Following transfection of the indicated *EDA* variants into HEK 293T cells (in serum-free OptiMEM medium), conditioned medium was used directly for Western blot (WB) analysis or first immunoprecipitated (IP) with beads coupled to anti-Flag M2 antibody, to EDAR-Fc, or to TACI-Fc. Beads were washed, eluted with 50 mM Na-citrate pH 2.7, and neutralized with Tris-HCl pH 9 as described previously (Schneider et al., 2014). The samples were analyzed by SDS-PAGE on 12% polyacrylamide mini gels under reducing conditions (30 mM dithiothreitol, with heating for 3 min at 70°C) or non-reducing conditions and analyzed by WB according to conventional protocols using anti-Flag M2 or anti-EDA Renzo-2 mAbs at 1 μg/ml followed by horseradish peroxidase-coupled anti-mouse secondary antibody, revelation with enhanced chemiluminescence (ECL) and either film exposure or signal acquisition with an iBright 750 imaging system (Fisher Scientific, Reinach, Switzerland).

### Surface expression and receptor-binding assay

HEK 293T cells modified by transfection of expression plasmids for full-length EDA (WT or variants), empty plasmid, and an EGFP expression plasmid at a ratio of 2/22/1 using the polyethyleneimide method were analyzed. Three days after the transfection, the cells were detached by pipetting, filtered through a nylon mesh, and stained in PBS, 5% FCS, 2 mM EDTA, 1 IU/ml of liquemin (Drossapharm, Basel, Switzerland) with EDAR-Fc at 50 μg/ml, followed by phycoerythrin-coupled goat anti-human IgG. The cells were analyzed by FACS with a CytoFLEX S apparatus (Beckman Coulter, Nyon, Switzerland).

### Statistical data analysis

Statistical analysis and graphical presentation of the data were performed using GraphPad Prism software version 9 (GraphPad Software Inc. La Jolla, United States). Groups were compared by one-way ANOVA, assuming equal standard deviation (SD) and comparing WT under all other conditions using Dunnett’s multiple comparison tests. Differences were considered significant when *p-*values were lower than 0.05.

## Results

**FIGURE 1:**
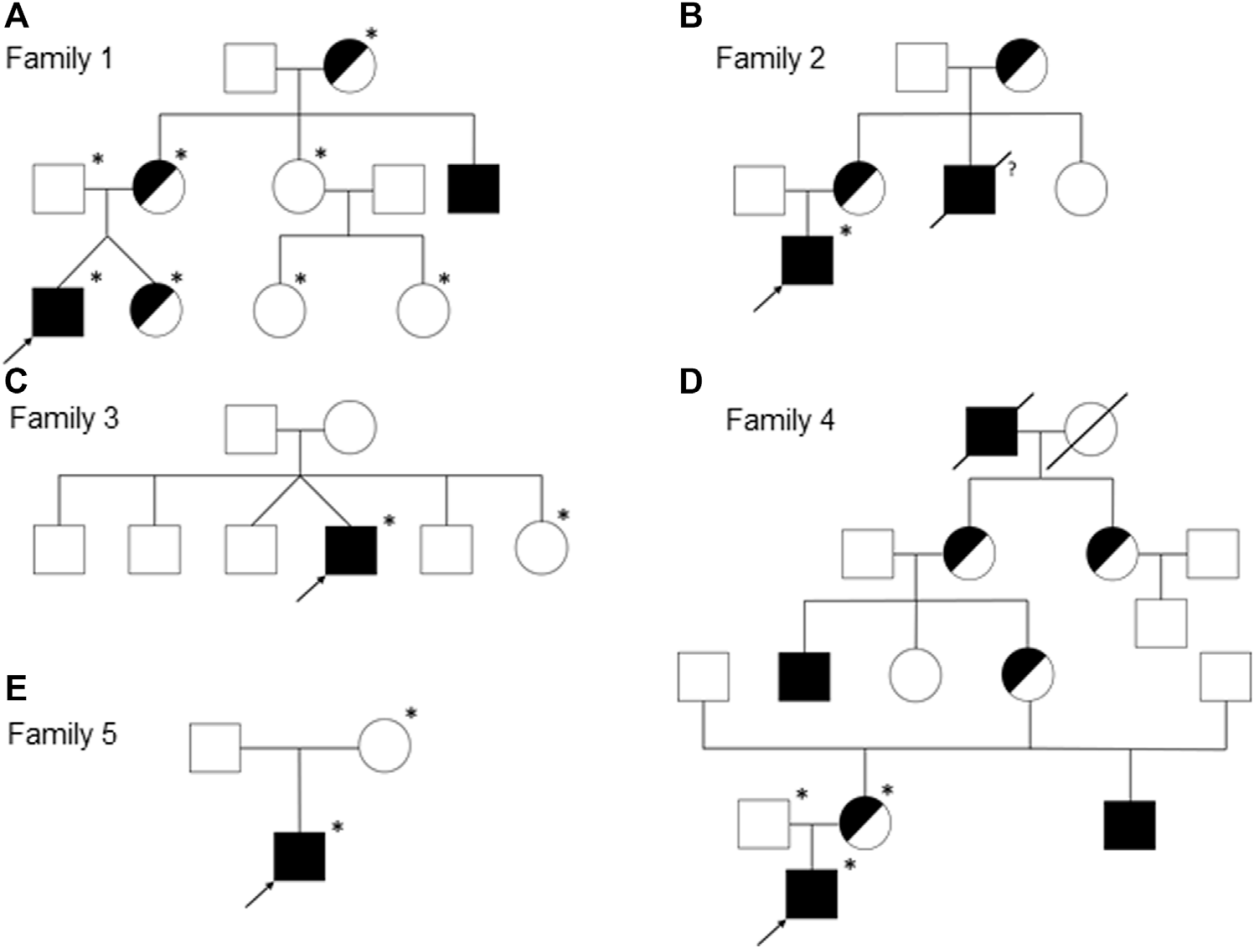
Pedigrees of the families 1 **(A)**, 2 **(B)**, 3 **(C)**, 4 **(D)**, and 5 **(E)**. Females are represented by circles, males by squares, presumed (heterozygous) carriers by half-shaded symbols, and subjects with fully expressed phenotypes by filled symbols. Arrows indicate the index patients of the respective families and asterisks the genotyped individuals. A slash through the symbol designates deceased persons and the question mark indicates an uncertain affection status.

**FIGURE 2:**
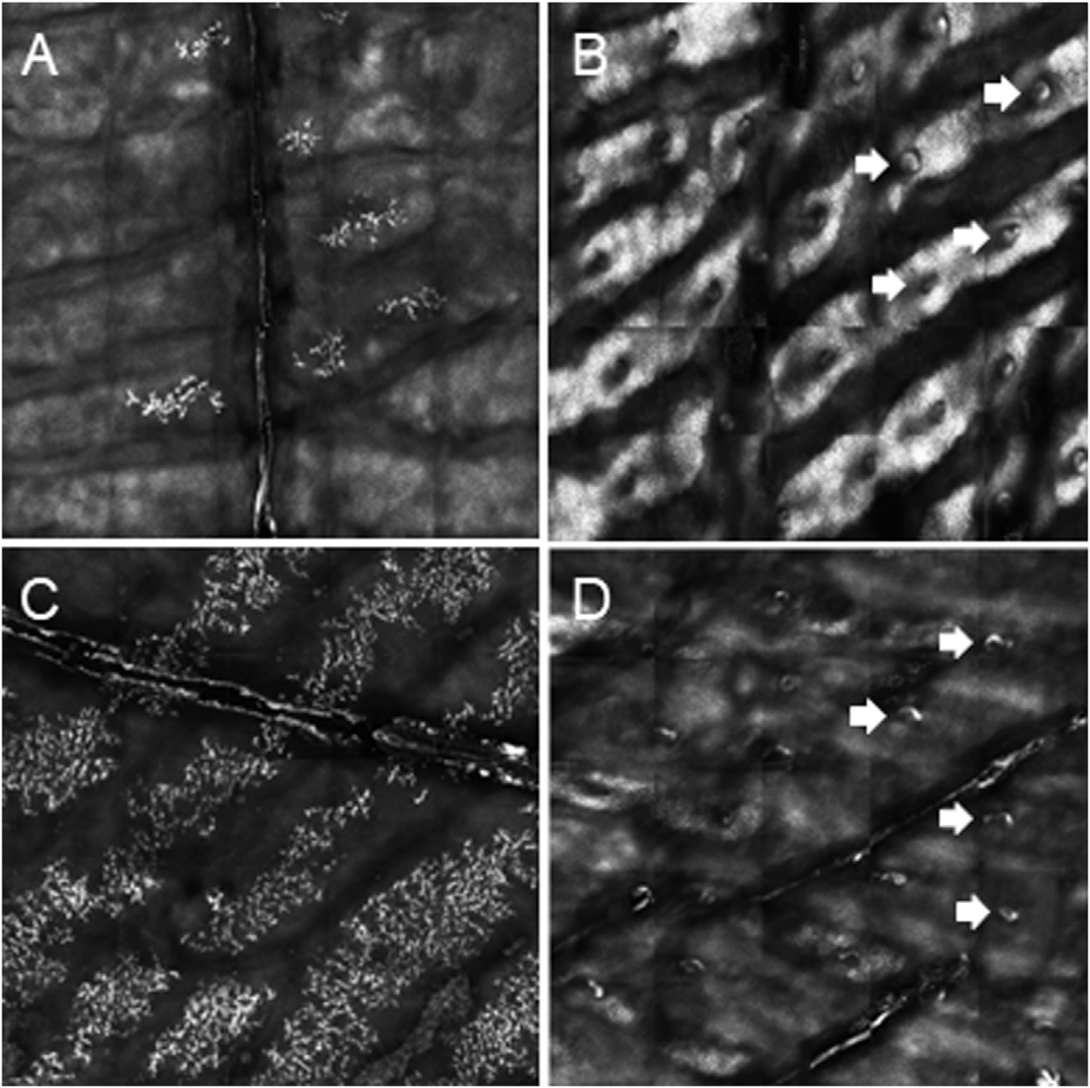
Determination of palmar sweat duct densities by confocal laser scanning microscopy (magnification of a representative area of 6.25 mm^2^). **(A)** Complete absence of sweat ducts in patient P1a. **(B)** Normal count and distribution of sweat ducts in his heterozygous sister P1b. **(C)** Lack of sweat ducts and distinct skin fissures in patient P3. **(D)** Reduced number of sweat pores in patient P4. White arrows indicate exemplarily some of the sweat pores.

We report on five families (families 1–5) with at least one member displaying classical symptoms of XLHED with variable phenotypic expression (the male index cases P1a, P2, P3, P4, and P5, and the female index case P1b, the twin sister of P1a, who also shows distinct symptoms). Health history assessment of the families 1, 2, and 4 revealed at least three generations of symptomatic individuals, showing an X-chromosomal inheritance pattern with females being less affected than males. In the index patients of families 3 and 5, the HED-related symptoms had emerged for the first time (Figure 1). Except for the female P1b, all of the patients suffered from skin issues (mainly dryness of the skin). Patient P4 was the only one with rather normal hair appearance, while the hair of the others was sparse or very sparse. Furthermore, all participants displayed very slow or even undetectable hair growth regarding eyelashes, eyebrows, and/or parts of body hair. For the adolescent and adult males, beard growth was rather normal and age-appropriate. All of the five boys had or still have to deal with eye issues like dry eyes or photophobia and with respiratory problems such as frequent bronchitis and nasal crusting. The number of deciduous and (where assessable) permanent teeth was markedly reduced in all patients and required (or will require) prosthodontic treatment. Episodes of heat intolerance have been experienced by all participants to varying extents and led to impairment of daily life aspects like outdoor sports or work. In line with their complete anhidrosis, patients P1a, P3, and P5 (respectively their parents) report about a severe heat intolerance and/or unexplained fever episodes (Table 1). Laser-scanning microscopy images document the complete absence of sweat ducts in patients P1a and P3 and, especially in case of P3, a very dry and fissured skin (Figures 2A,C). Patient P1b displayed a normal sweat duct density (Figure 2B) but an abnormal pilocarpine-induced sweat volume of only 8 µl (normal range between 29 and 93 μl, Schneider et al., 2011). She indicated that she had suffered from heat intolerance in her childhood but did not do so anymore. P2 self-assessed his heat sensitivity as rather low and reported to be able to sweat in various body areas, which corresponds with his reduced sweat pore density and pilocarpine-induced sweat volume (assessed at the age of 6 years; Schneider et al., 2011). Interestingly, patient P4 reported to be rather mildly affected by heat intolerance, although no sweat could be collected on his forearm upon stimulation with pilocarpine. Confocal laser-scanning microscopy revealed the presence of about 200 sweat ducts/cm^2^, but with an irregular distribution among the areas analyzed (Figure 2D). Like most HED patients, all affected subjects investigated in this study were slender or even underweight. Allergies were found in four, abnormalities of the mammillae in two patients (poly- and athelia; Table 1).

**TABLE 1:** Phenotypic features.

Code	Age in years	Skin	Scalp hair	Eyebrows/- lashes	Eyes	Respiratory problems	Current dental status	Reported heat sensitivity/intolerance	Body areas reportedly capable of sweating	Sweat ducts per cm^2^	Sweat volume in µL	Other
P1^a^	16	Dry	Sparse, very light	Both sparse	Dry	No^a^	4 d.t. + 1 p.t.	Yes	None	0	0	Allergies
P1^b^	16	Unremarkable	Rather sparse, light	Both sparse	Unremarkable	No	3 d.t. + 20 p.t.	No^b^	All-over	408	8	Allergies
P2	17	Dry, fissured	Sparse, light	Sparse/unremarkable	Mild photophobia	No^c^	9 d.t. + 10 p.t.	Yes	Hands, feet, genital area, bends of the elbows, hollows of the knees, armpits	62^d^	11^d^	Allergies, polythelia
P3	28	Very dry	Very sparse, very light	Both very sparse	Photophobia	Recurrent sinusitis, nasal crusting	2 d.t. + 0 p.t.	Yes	Feet	0	0	Bilateral athelia, asthenic habitus
P4	8	Dry, neurodermatitis	Rather normal, light	Very sparse/sparse	Unremarkable^e^	No^f^	10 d.t. + n.a.	Yes (mild)	Armpits, back	200	0	Allergies
P5	3	Dry, partly eczematous, aplasia cutis congenita (healed)	Sparse	Absent/sparse	Lacrimal duct stenosis	Nasal crusting	6 d.t. + n.a.	Episodes of hyperthermia in early infancy	None	n.a.	0	Impaired weight gain, sleep apnoea in first months of life

Abbreviations: d.t., deciduous teeth; p.t., permanent teeth; n.a., not assessed.

^a^ Except for bronchitis and nasal crusting in infancy.

^b^ But in infancy.

^c^ Except for recurrent laryngitis and nasal crusting in infancy.

^d^ Assessed at the age of 6 years (Schneider et al., 2011).

^e^ Except for recurrent conjunctivitis in early infancy.

^f^ Except for recurrent nosebleeds in early infancy.

Genetic diagnosis of the index patients was attempted by sequencing the most relevant HED-associated genes (Sanger and multi-gene panel sequencing, respectively), which revealed five different *EDA* variants of uncertain significance as indicated by deviating conclusions of various variant effect prediction tools (Table 2). Segregation analysis and genetic testing of close relatives (siblings, parents, grandparents, and cousins) were performed, wherever possible (Figure 1).

**TABLE 2:** *EDA* genotypes of affected individuals and assessments of variant effect prediction tools.

Code	*EDA* variant	Changes at the amino acid level	MutationTaster	Fruitfly	Provean	PolyPhen-2	SIFT	Fathmm	ACMG classification
P1	c.924+7A > G	—	Disease-causing	No splice site change predicted	—	—	—	—	4—likely pathogenic
P2	c.527G > T^a^	p.Gly176Val	Disease-causing	No splice site change predicted	Neutral	Probably damaging	Tolerated	Damaging	4—likely pathogenic
P3^b^	c.1166del	p.Pro389LeufsX27	Polymorphism	—	Neutral	—	—	—	5—pathogenic
P4	c.374C > G^c^	p.Ser125Cys	Polymorphism	—	Neutral	Benign	Tolerated	Damaging	4—likely pathogenic
P5	c.1174T > C^d^	p.Ter392GlnfsX30	Polymorphism	—	—	—	—	—	5—pathogenic

a Schneider et al.(2011)

b Additionally heterozygous carrier of the *WNT10A* variant c.682T > A (p.Phe228Ile).

c Guazzarotti et al.(2015).

d Hashiguchi et al.(2003).

**FIGURE 3:**
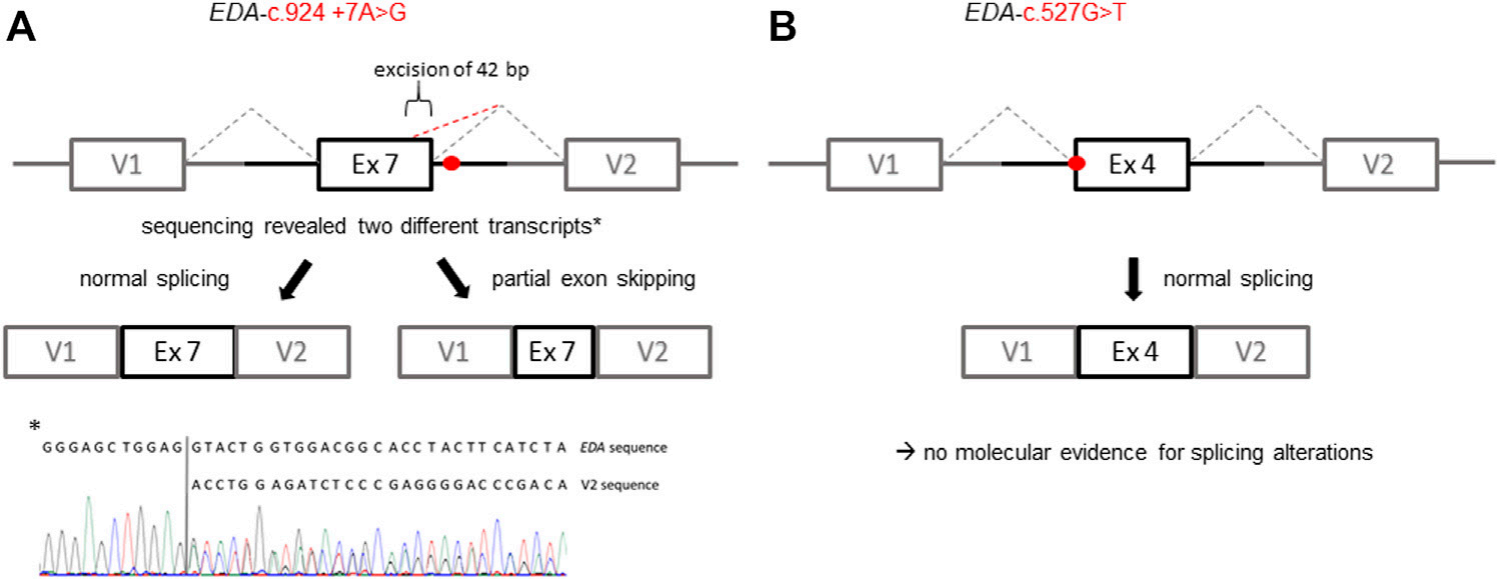
Schematic illustration of the exon trapping assays displaying the potential effects of the *EDA* variants on splicing. **(A)** Variant *EDA*-c.924+7A > G spawns two different transcripts *in vitro* indicated by a normally spliced WT sequence and additionally a partially skipped exon (truncation of 42 base pairs; GenBank accession number: ON600878). **(B)** With regard to the variant *EDA*-c.527G > T there was no evidence for variant-induced alternative splicing, as only the WT sequence was detectable. V1, vector exon 1; V2, vector exon 2.

**FIGURE 4:**
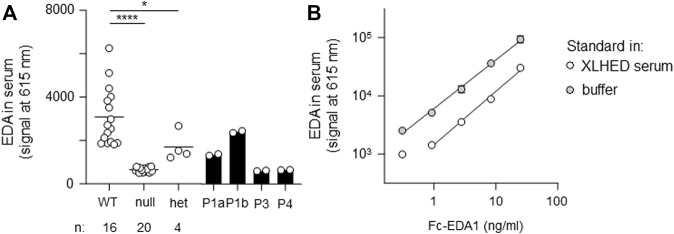
AlphaLISA measurements of circulating EDA in serum samples. **(A)** Probes were analyzed in duplicates. The average value is displayed for wild-type (WT, *n* = 16), null mutations (null, *n* = 20), and heterozygous variants (het, *n* = 4). Individual values are shown for patients P1a, P1b, P3, and P4. **(B)** Recombinant Fc-EDA1 standard was measured at the indicated concentrations either in assay buffer or in serum of an XLHED individual with a null mutation.

All affected and tested members of family 1 were either heterozygous (female) or hemizygous (male) carriers of the *EDA* variant c.924+7A > G. This variant has not been described in the literature yet, but there are known splice site variants nearby and even in the adjacent position (van der Hout et al., 2008). Diagnostic panel sequencing revealed no other potentially pathogenic variants in HED-related genes. The variant lies close to the splice donor site of intron 7, which is located in the TNF domain. Although the deviation is not directly in the consensus sequence, the program MutationTaster assessed the variant as potentially disease-causing due to marginally changed splicing (Table 2). To elucidate the potential effect on splicing, an exon trapping assay using mini-gene constructs (WT and mutated, including part of intron 6, exon 7, and part of intron 7) was performed. Sequencing of WT RT-PCR product revealed normal splicing (data not shown), while the *EDA*-c.924+7A > G sample yielded two different transcripts, the normally spliced sequence and one with a shortened exon 7 (excision of 42 base pairs; Figure 3A). Circulating EDA levels in the blood sera were measured by AlphaLISA. The samples from 16 healthy individuals (WT) and 20 XLHED patients with well-known pathogenic variants (null mutations) served as positive and negative controls, respectively. Congruent with the result of the splice site assay, patient P1a displayed only slightly reduced levels of EDA compared with WT while heterozygous patient P1b had circulating levels of EDA comparable to those of healthy controls (Figure 4).

**FIGURE 5:**
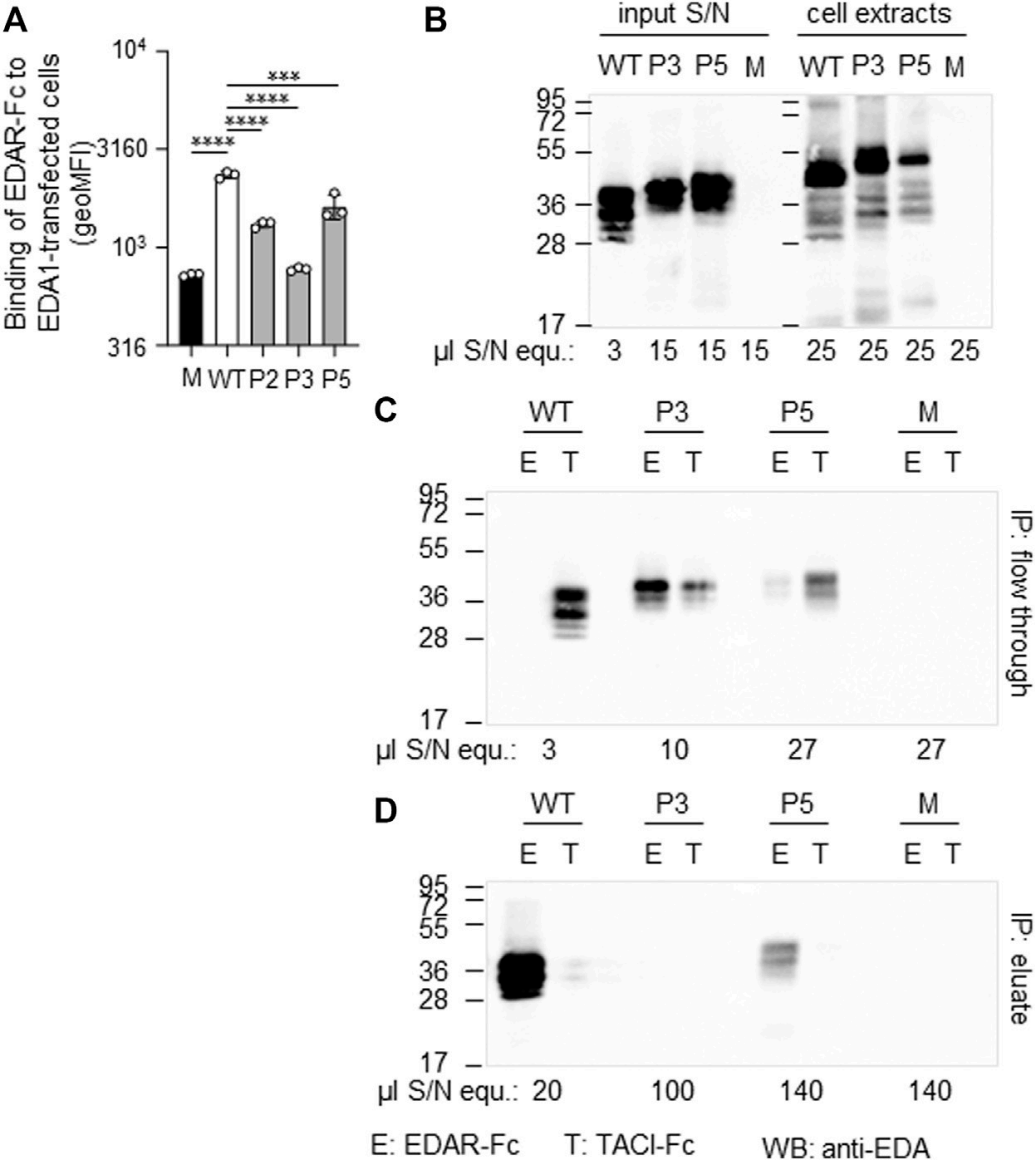
Expression and receptor-binding ability of EDA variants p. Gly175Val (P2), p.Pro389LeufsX27 (P3) and p.Ter392GlnfsX30 (P5). **(A)** HEK 293T cells expressing full-length *EDA* or variants thereof were monitored by FACS for their capability of binding to EDAR-Fc, indicated as geometric mean of fluorescence intensity (geoMFI). Mock transfectants (empty plasmid) were used as negative control (M, mock). **(B)** Cell extracts (right hand side) and conditioned supernatants (left hand side) of transfected HEK 293T cells were analyzed by WB with anti-EDA mAb Renzo-2. Amounts of sample loaded are shown as µl equivalent of conditioned medium (µl S/N equ). **(C,D)** Conditioned supernatants of HEK 293T-cells were immunoprecipitated with immobilized EDAR-Fc (E) or TACI-Fc (T) as control. The unbound (flow through) and retained (eluate) fractions were analyzed by WB with anti-EDA mAb Renzo-2. The respective volumes of samples loaded are shown as µl equivalent of conditioned medium. The migration positions of molecular weight markers (in kDa) are indicated on the left.

A hemizygous missense variant c.527G > T (p.Gly176Val) affecting the first base of exon 4 was detected in patient P2. Other family members likely to carry the same variant (his mother, grandmother, and a maternal uncle who died one day after birth) were not available for genetic testing (Figure 1B). Although the adjacency of the variant to the splice acceptor consensus sequence makes an impact on splicing possible, the exon trapping assay (mini-gene constructs containing part of intron 3, exon 4, and part of intron 4) showed no difference between the WT control and the mutated construct (Figure 3B). When full-length EDA1-Gly176Val was expressed in HEK 293T cells, these cells readily bound to recombinant EDAR-Fc, albeit less efficiently than WT (Figure 5A), indicating that the protein can be expressed in a form capable of binding to its receptor.

Patient P3 displayed a hemizygous *EDA* deletion, c.1166del (p.Pro389LeufsX27), resulting in an exchange of the last two amino acids and an exotic C-terminal tail of 26 additional amino acids. Furthermore, patient P3 is a heterozygous carrier of the *WNT10A* variant c.682T > A (p.Phe228Ile) known to evoke tooth agenesis or even HED when present in homozygous or compound-heterozygous states. Although only his sister was available for genetic testing, it is likely that the *EDA* variant arose *de novo*, as none of his family members (including his four brothers, one sister, and the mother) had any specific symptoms. The protein could be produced but not as efficiently as WT. Both full-length EDA in cell extracts and the cleaved form in cell supernatants were larger than wild-type EDA, in line with the presence of the C-terminal extension (Figure 5B). In addition, it was unable to bind to EDAR in a co-immunoprecipitation experiment (Figures 5C,D). When expressed as a membrane-bound protein in HEK 293T cells, p.Pro389LeufsX27 was similarly incapable of binding to EDAR-Fc (Figure 5A), suggesting that this variant is inactive.

**FIGURE 6:**
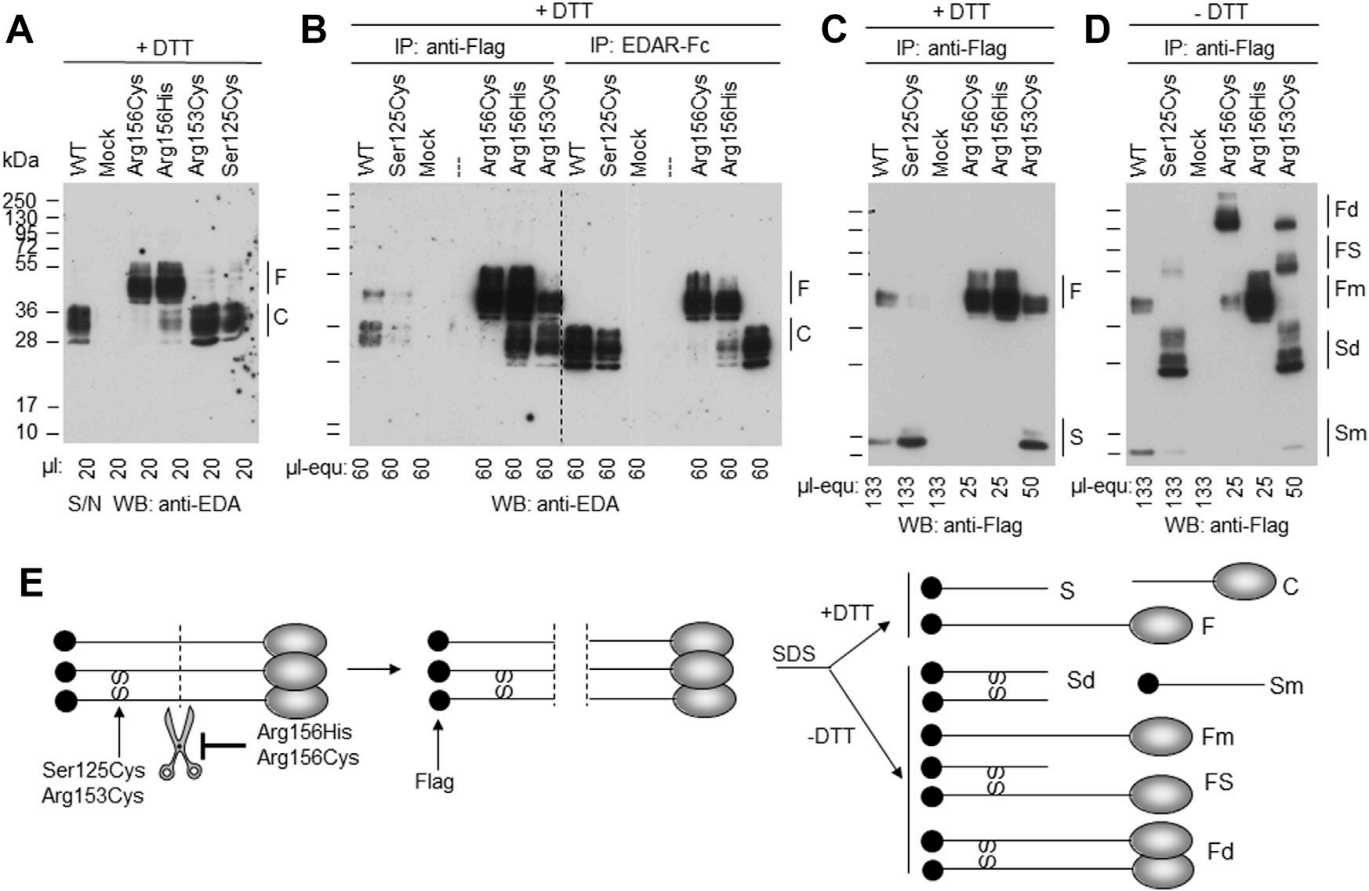
EDA1-Ser125Cys is secreted and processed at the FUR as efficiently as WT but induces formation of disulfide bridges. Conditioned supernatants (S/N) of HEK 293T cells transiently expressing a soluble form of Flag-EDA (starting as Ser66 after the TM), with or without XLHED-causing variants in the stalk region or FUR, were either used directly or immunoprecipitated with immobilized anti-Flag mAb or with immobilized EDAR-Fc. The samples were analyzed by WB with either anti-EDA mAb Renzo-2 or anti-Flag mAb M2 as indicated. Renzo-2 and M2 recognize the C-terminal and N-terminal portions of the constructs, respectively. **(A)** Cell supernatants analyzed by Western blot with anti-EDA under reducing (+DTT) conditions. **(B)** Cell supernatants analyzed by WB with anti-EDA under reducing (+DTT) conditions after immunoprecipitation with anti-Flag or with EDAR-Fc. The amount loaded is indicated as µl equivalent of conditioned medium (µl-equ). **(C)** Same anti-Flag immunoprecipitation as shown in panel B, except that the blot was revealed with anti-Flag and that amounts loaded in each lane were adjusted as indicated. **(D)** Same as panel C, but with samples that were not reduced (- DTT). **(E)** Schematic drawing of EDA with N-terminal Flag tag (black circle) and C-terminal receptor-binding domain (gray ovals forming 3-mers under native conditions, according to protein data bank entry 1RJ7). Scissors represent furin or furin-like activities. SS represent disulfide bonds observed in EDA variants p. Ser125Cys, p. Arg153Cys and p. Arg156Cys. Schemes on the left and in the middle represent unprocessed and cleaved native EDA, respectively. Schemes on the right represent denatured (SDS) protein in the presence (+DTT, top) or absence (-DTT, bottom) of reducing agent to yield fragments with or without disulfide bonds. F, full length; C, cleaved; S, stalk; Fd, disulfide-linked dimer of full EDA; FS, full EDA disulfide-linked to a stalk; Fm, non-disulfide-linked monomer of full EDA; Sd, disulfide-linked dimer of stalk; Sm, non-disulfide-linked monomer of stalk; SS, inter-chain disulfide bonds.

The EDA variant of patient P4, c.374C > G (p.Ser125Cys), affects the stalk region of EDA between TM and FUR. A construct in which the ICD and the TM of WT EDA were replaced by a secretion signal and a Flag tag was expressed in parallel with EDA1-Ser125Cys or with variants known to affect one (p.Arg153Cys) or both (p.Arg156His and p.Arg156Cys) of the two consensus FUR. WB analysis with an anti-EDA antibody recognizing the C-terminal part of EDA revealed that the variants p.Arg156Cys and p.Arg156His, as expected, were not or poorly cleaved at the FUR, while WT EDA was processed to a smaller form. EDA1-Arg153Cys was also processed efficiently, indicating that in HEK 293T cells this can occur at the second furin cleavage site. EDA1-Ser125Cys was processed similarly to WT, demonstrating the absence of a processing defect (Figure 6A). Unprocessed EDA was detected in all cases, at high or intermediate levels for FUR variants or at low to very low levels for WT and p.Ser125Cys, respectively (Figure 6B, left). Moderate amounts of co-immunoprecipitating cleaved EDA that actually should not contain the Flag tag anymore can be explained if only one or two EDA molecules in the 3-mer have been processed (Figure 6B, left). All forms of EDA containing the C-terminal domain could bind to EDAR-Fc (Figure 6B, right). Solely unprocessed EDA and cleaved, Flag-containing N-terminal fragments were detectable by WB anti-Flag (Figure 6C). For WT EDA and p.Arg156His, migration profiles were identical under reducing and non-reducing conditions, indicating the absence of inter-chain disulfide bridges (Figures 6C,D). However, the EDA1 variants p.Ser125Cys, p.Arg153Cys, and p.Arg156Cys, in which cysteine residues were generated by the alterations, led to the following products: dimers of full length EDA1 (in case of the uncleavable EDA1-Arg156Cys or the difficult-to-cleave p.Arg153Cys), dimers of the N-terminal fragment (p.Ser125Cys and p.Arg153Cys), or heterodimers between cleaved and uncleaved EDA for those variants, where processing still took place (p.Ser125Cys and p.Arg153Cys, Figure 6D). Although the *in vitro* analyses of overexpressed EDA1-Ser125Cys showed abnormal disulfide bridge formation, this did not translate into obvious defects of the molecule explaining the XLHED phenotype. Nevertheless, circulating EDA was undetectable in the serum of P4, indicating an inhibited expression or processing at the endogenous level (Figure 4).

The variant c.1174T > C (p.Ter392GlnfsX30) of patient P5 disrupts the original termination codon, leading to a prolongation of the protein by 29 amino acids, and similar to EDA variant p.Pro389LeufsX27, its expression was lower than WT EDA. However, p.Ter392GlnfsX30 did not abolish binding to EDAR (Figures 5A,C,D), suggesting that this variant may still have some activity *in vivo*. Unfortunately, no serum of subject P5 was available for measurement of circulating EDA.

## Discussion

The present study aimed at elucidating the genetic background of five familial XLHED cases with *EDA* variants of yet unknown significance. The second aim was to find potential genotype–phenotype correlations.

A critical aspect of many studies (including the current one) is the subjectivity of patient-reported symptoms, such as individual heat intolerance or skin problems. Isolated quantitative Likert-scale surveys, where the respondent is asked to rate his or her answer (e.g., 0 = never, 1 = rarely, 2 = sometimes, 3 = quite often, and 4 = often), have already been conducted regarding symptom severity, quality of life, and psychological distress among patients with ectodermal dysplasia (Pavlis et al., 2010; Kohli et al., 2011; Saltnes et al., 2017). However, more standardized questionnaires covering the most relevant aspects of XLHED, developed by a multidisciplinary team of experts and translated into different languages, would likely improve global comparability among the patients. It might also be useful to estimate the impact of XLHED on individuals with hypomorphic variants and on female carriers, which might be relevant for the question whether future medical treatment should be considered for them, too. More objective parameters for the classification of XLHED phenotypes are the number of sweat glands and the quantity of pilocarpine-induced sweating. Nevertheless, standard operating procedures and large data collections considering age, sex, and body surface are needed for the definition of reference values.

A definite and genetically confirmed diagnosis often means great relief for the patients and their families and is essential for profound statements regarding inheritance pattern, prognosis, and therapeutic options. With regard to the current prenatal therapy trial, a definite diagnosis of XLHED (either by prenatal tooth germ sonography or by detection of a pathogenic *EDA* variant in the fetus) belongs to the inclusion criteria. However, unambiguous classification, especially of novel or very rare sequence variants can be challenging. Pedigree analysis is an important statistical technique to examine whether a gene variant co-segregates with a certain trait, but the informative value strongly depends on the pedigree size and the availability of genetic material from relevant family members. Furthermore, the co-occurrence of variants and certain features might also be coincidental and, as a matter of course, this analysis is not applicable for *de novo* variants.

In the current study, we were able to establish unambiguous genotype–phenotype relationships for some but not all of the *EDA* variants studied, probably due in part to limitations or inadequacy of *in vitro* assays. For example, overexpressed EDA1-Pro389LeufsX27 could not bind to its receptor in two different *in vitro* assays. Moreover, the EDA protein was undetectable in serum of patient P3. These two criteria fit with and could explain clinical observations of XLHED symptoms and anhidrosis. This also indicates that the mere fact to successfully overexpress EDA may not necessarily correlate with protein expression at the endogenous level *in vivo*, and that measuring circulating EDA is probably a good assessment of endogenous protein expression. Results obtained with EDA1-Ser125Cys corroborate this hypothesis, as the only correlation with clinical manifestation was the absence of circulating EDA. It is possible that the exotic disulfide bridge detected in the overexpressed EDA1-Ser125Cys may affect expression or processing at the endogenous level but not upon overexpression. The availability of EDA1-Ter392GlnfsX30 in P5 may be clearly reduced compared to WT, but the protein could still be processed and bind to EDAR *in vitro*. Probably EDA-Ter392GlnfsX30 has reduced affinity for EDAR relative to WT, but even if this was the case it would not exclude that residual activity could be functionally relevant *in vivo*. Variant c.924+7A > G in intron 7 of P1a and P1b does not affect the consensus splicing sequence but induced aberrant splicing in an exon trapping assay *in vitro* that would result in a deletion of 14 amino acids in the TNF domain, should this happen *in vivo*. Circulating EDA levels were reduced but detectable in P1a. Clinical manifestation of XLHED, and anhidrosis could be explained if circulating EDA is the mis-spliced protein or if the mis-spliced form could exert a dominant-negative effect on the WT form. Finally, EDA1-Gly176Val (c.527G > T) of P2 could be expressed from the cDNA and bind to EDAR. In addition, according to current knowledge about functional domains of EDA, there is no obvious reason why this variant should affect processing, receptor binding, multimerization, or interactions with proteoglycans. It, however, affects the first nucleotide of exon 4. In a comprehensive *in silico* analysis of all human splice sites (concerning 327,293 exons across 81,814 different transcripts among 20,345 human genes), this position is 47.8% G vs. only 11.5% T (Ma et al., 2015). Despite the fact that the exon trapping assay was insensitive to this variant, it is difficult to exclude that it could decrease protein expression *in vivo*, which would fit with the residual sweating activity reported by P2 and measured with respect to pilocarpine-induced sweating on the forearm. It is also in agreement with *in vitro* data showing that the mutant protein is not biochemically inactive. As a general conclusion, *in vitro* assays performed in this study can be useful to link genotype with phenotype but appear to underestimate the pathogenic impact of some variants. Measurement of circulating EDA levels can provide useful additional information to assess pathogenicity. Finally, although *in vitro* tests could be used to exclude some patients from the evaluation of clinical efficacy of a prenatal protein replacement therapy, such tests should not be used to deny access to treatment of unborn patients diagnosed with oligodontia and carrying any individual *EDA* variant.

*In silico* prediction algorithms as well as functional and biochemical studies are important tools to interpret the effect of a variant and, advantageously, do not necessarily require biological material from patients. Still, in some cases these approaches do not reflect *in vivo* conditions and are unable to unequivocally prove causality. The measurement of circulating EDA levels provides an independent criterion to ascertain that an EDA variant is the cause of disease. Indeed, a circulating form of EDA is not only present during embryogenesis but also in the adult human sera (Podzus et al., 2017). It can reasonably be assumed that the absence of circulating EDA is synonymous with a null mutation status, but not necessarily vice versa, as the protein might be detectable but functionally impaired. Importantly, this potential biomarker of null mutations is independent of changes in the phenotype that could be induced by a successful protein replacement therapy and could therefore also be monitored after treatment, once recombinant Fc-EDA1 has been fully metabolized and eliminated. Systematic studies with larger numbers of patients and controls are needed to assess the extent to which levels of circulating EDA correlate with a disease phenotype.

The improvement of diagnostic processes and the extension of knowledge about XLHED-causing mechanisms will enable more families to benefit from medical treatments in the future.

## Data Availability

The datasets presented in this study can be found in online repositories. The names of the repository/repositories and accession number(s) can be found from the following: NCBI GenBank, ON600878.
