# Erratum: Functional and clinical analysis of five *EDA* variants associated with ectodermal dysplasia but with a hard-to-predict significance

**DOI:** 10.3389/fgene.2023.1281631

**Published:** 2023-08-31

**Authors:** Frontiers Production Office

**Affiliations:** Frontiers Media SA, Lausanne, Switzerland

**Keywords:** X-linked hypohidrotic ectodermal dysplasia, Ectodysplasin A, variants of uncertain significance, *in silico* analysis, functional studies, serum EDA concentration, genotype-phenotype correlation

Due to a production error, a formatting error was made. In Table 1, in the third line of the column “Sweat volume in µL” should be “11^d^” instead of “114”. The corrected table appears below.

**TABLE 1:** Phenotypic features.

Code	Age in years	Skin	Scalp hair	Eyebrows/- lashes	Eyes	Respiratory problems	Current dental status	Reported heat sensitivity/intolerance	Body areas reportedly capable of sweating	Sweat ducts per cm^2^	Sweat volume in µL	Other
P1^a^	16	Dry	Sparse, very light	Both sparse	Dry	No^a^	4 d.t. + 1 p.t.	Yes	None	0	0	Allergies
P1^b^	16	Unremarkable	Rather sparse, light	Both sparse	Unremarkable	No	3 d.t. + 20 p.t.	No^b^	All-over	408	8	Allergies
P2	17	Dry, fissured	Sparse, light	Sparse/unremarkable	Mild photophobia	No^c^	9 d.t. + 10 p.t.	Yes	Hands, feet, genital area, bends of the elbows, hollows of the knees, armpits	62^d^	11^d^	Allergies, polythelia
P3	28	Very dry	Very sparse, very light	Both very sparse	Photophobia	Recurrent sinusitis, nasal crusting	2 d.t. + 0 p.t.	Yes	Feet	0	0	Bilateral athelia, asthenic habitus
P4	8	Dry, neurodermatitis	Rather normal, light	Very sparse/sparse	Unremarkable^e^	No^f^	10 d.t. + n.a.	Yes (mild)	Armpits, back	200	0	Allergies
P5	3	Dry, partly eczematous, aplasia cutis congenita (healed)	Sparse	Absent/sparse	Lacrimal duct stenosis	Nasal crusting	6 d.t. + n.a.	Episodes of hyperthermia in early infancy	None	n.a.	0	Impaired weight gain, sleep apnoea in first months of life

Abbreviations: d.t., deciduous teeth; p.t., permanent teeth; n.a., not assessed.

^a^ Except for bronchitis and nasal crusting in infancy.

^b^ But in infancy.

^c^ Except for recurrent laryngitis and nasal crusting in infancy.

^d^ Assessed at the age of 6 years (**Schneider et al., 2011**).

^e^ Except for recurrent conjunctivitis in early infancy.

^f^ Except for recurrent nosebleeds in early infancy.

The publisher apologizes for this mistake. The original version of this article has been updated.

