# Supplementary material for: Functional and clinical analysis of five EDA variants associated with ectodermal dysplasia but with a hard-to-predict significance
Source: Front Genet. 2022 Jul 18;13:934395. doi: 10.3389/fgene.2022.934395 (PMC9339965; doi:10.3389/fgene.2022.934395)
Supplement: Supplementary file 1 [file Table1.DOCX]

Supplementary Material

Table S1: List and description of the plasmids used in the study

| **Plasmid** | **Designation** | **Protein encoded/insert** | **Backbone** |
| --- | --- | --- | --- |
| ps015 | Empty | Empty | PCR3 |
| ps905 | Flag-EDA1 Ser66 Arg153Cys | HA signal-Flag-GPGQVQLQVD-hEDA1 Arg153Cys (66-391) | PCR3 |
| ps930 | hEDAR-hFc | hEDAR (1-183)-VD-hIgG1 (245-470) | PCR3 |
| ps937 | Flag-EDA1 Ser66 WT | HA signal-Flag-GPGQVQLQVD-hEDA1 (66-391) | PCR3 |
| ps1043 | Flag-EDA1 Ser66 Arg156Cys | HA signal-Flag-GPGQVQLQVD-hEDA1 Arg156Cys (66-391) | PCR3 |
| ps1109 | Flag-EDA1 Ser66 Arg156His | HA signal-Flag-GPGQVQLQVD-hEDA1 Arg156His (66-391) | PCR3 |
| ps3786 | hEDAR-PS-hFc | hEDAR (1-183)-VD-PreSci-hIgG1 (245-470) | PCR3 |
| ps3825 | hTACI-Fc | Ig signal-hTACI (31-110)-VD- hIgG1 (245-470) | PCR3 |
| ps4036 | Flag-EDA1 Ser66 Ser125Cys | HA signal-Flag-GPGQVQLQVD-hEDA1 Ser125Cys (66-391) | PCR3 |
| ps1752 | hEDA1 full WT | hEDA1 (1-391) | PCR3 |
| ps4066 | hEDA1 full Pro389LeufsX27 | hEDA1 (1-389)-LHPRFPPFCLCPCPFPGFGSQDSQNL | PCR3 |
| ps4067 | hEDA1 full Ter392GlnfsX30 | hEDA1 (1-391)-QIPPILPLSVPLPWVWEPGLPEPLSAAVE | PCR3 |
| ps4178 | hEDA1 full Gly176Val | hEDA1 Gly176Val (1-391) | PCR3 |
| / | pSPL3 EDA c.924+7A>G | Part of intron 6, exon 7, part of intron 7 | pSPL3 |
| / | pSPL3 EDA c.527G>T (Gly176Val) | Part of intron 3, exon 4, part of intron 4 | pSPL3 |

HA signal=MAIIYLILLFTAVRG Ig signal=METDTLLLWVLLLLWVPGVHG

Flag=DYKDDDDK PreSci=LEVLFQGP
