# Supplementary figures and images for: Functional and clinical analysis of five EDA variants associated with ectodermal dysplasia but with a hard-to-predict significance
Source: Front Genet. 2022 Jul 18;13:934395. doi: 10.3389/fgene.2022.934395 (PMC9339965; doi:10.3389/fgene.2022.934395)

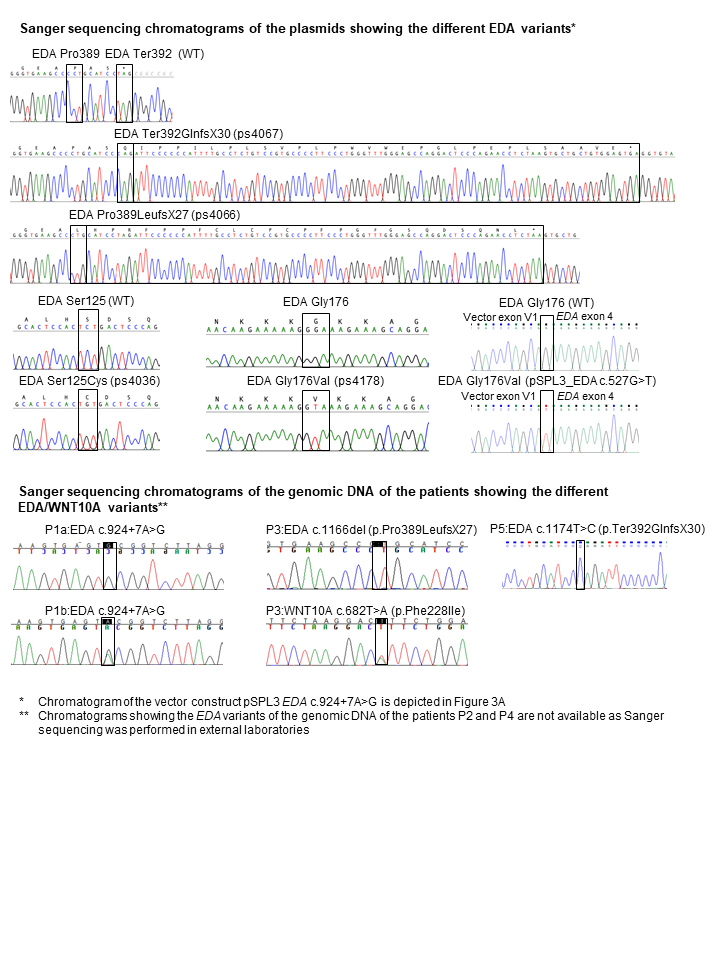

Supplement: Supplementary file 2 [file Image1.TIF]
